# Supplementary material for: Subjects are not all alike: Eye-tracking the agent preference in Spanish
Source: PLoS One. 2022 Aug 3;17(8):e0272211. doi: 10.1371/journal.pone.0272211 (PMC9348668; doi:10.1371/journal.pone.0272211)
Supplement: S1 Table — Online norming study results showing the mean ratings for strongly related noun-noun pairs. (DOCX) [file pone.0272211.s001.docx]

**S1 Table. Mean ratings of strongly-related nouns.**

| **Noun 1** | **Noun 2** | **Mean rating** |
| --- | --- | --- |
| *anciano* ‘old man’ | *bastón* ‘cane’ | 4,69 |
| *arquero* ‘archer’ | *flecha* ‘arrow’ | 4,98 |
| *barbero* ‘barber’ | *barba* ‘beard’ | 4,87 |
| *barrendero* ‘sweeper’ | *escoba* ‘broom’ | 4,87 |
| *bebé* ‘baby’ | *biberón* ‘baby bottle’ | 4,87 |
| *bombero* ‘firefighter’ | *camión de bomberos* ‘firetruck’ | 4,98 |
| *canario* ‘canary’ | *jaula de* *pájaro* ‘bird cage’ | 4,7 |
| *cantante* ‘singer’ | *micrófono* ‘microphone’ | 4,81 |
| *carpintero* ‘carpenter’ | *madera* ‘wood’ | 4,76 |
| *cartero* ‘mailman’ | *buzón* ‘mailbox’ | 4,92 |
| *científico* ‘scientist’ | *microscopio* ‘microscope’ | 4,63 |
| *conductor* ‘driver’ | *coche* ‘car’ | 4,85 |
| *conejo* ‘rabbit’ | *zanahoria* ‘carrot’ | 4,7 |
| *costurera* ‘seamstress’ | *aguja de coser* ‘sewing needle’ | 4,92 |
| *electricista* ‘electrician’ | *bombilla* ‘light bulb’ | 4,72 |
| *escritor* ‘writer’ | *máquina de escribir* ‘typewriter’ | 4,69 |
| *explorador* ‘explorer’ | *mapa* ‘map’ | 4,9 |
| *gallina* ‘hen’ | *huevo* ‘egg’ | 4,96 |
| *leñador* ‘lumberjack’ | *tronco* ‘log’ | 4,9 |
| *limpiadora* ‘cleaner’ | *fregona* ‘mop’ | 4,63 |
| *marinero* ‘sailor’ | *barco* ‘ship’ | 4,94 |
| *músico* ‘musician’ | *violín* ‘violin’ | 4,69 |
| *oculista* ‘oculist’ | *gafas* ‘glasses’ | 4,9 |
| *pájaro* ‘bird’ | *nido* ‘nest’ | 4,96 |
| *párroco* ‘priest’ | *iglesia* ‘church’ | 4,94 |
| *peluquera* ‘hairdresser’ | *peine* ‘comb’ | 4,92 |
| *percusionista* ‘drummer’ | *tambor* ‘drum’ | 4,81 |
| *perro* ‘dog’ | *hueso* ‘bone’ | 4,8 |
| *pescador* ‘fisherman’ | *caña de pescar* ‘fishing pole’ | 4,87 |
| *piloto* ‘pilot’ | *avión’* airplane’ | 4,96 |
| *preso* ‘inmate’ | *esposas* ‘handcuffs’ | 4,63 |
| *ratón* ‘mouse’ | *queso* ‘cheese’ | 4,72 |
| *reina* ‘queen’ | *corona* ‘crown’ | 4,9 |
| *sacerdote* ‘priest’ | *cruz’* cross’ | 4,74 |

Online norming study results showing the mean ratings for strongly related noun-noun pairs.
