# Supplementary material for: Subjects are not all alike: Eye-tracking the agent preference in Spanish
Source: PLoS One. 2022 Aug 3;17(8):e0272211. doi: 10.1371/journal.pone.0272211 (PMC9348668; doi:10.1371/journal.pone.0272211)
Supplement: S2 Table — Online norming study results showing the mean ratings for weakly-related noun-noun pairs. (DOCX) [file pone.0272211.s002.docx]

**S2 Table. Mean ratings of weakly-related nouns.**

| **Noun 1** | **Noun 2** | **Mean rating** |
| --- | --- | --- |
| *asesino* ‘murderer’ | *microscopio* ‘microscope’ | 0,67 |
| *atleta* ‘athlete’ | *mapa* ‘map’ | 0,67 |
| *bebé* ‘baby’ | *barba* ‘beard’ | 0,27 |
| *cachorro* ‘puppy’ | *nido* ‘nest’ | 0,72 |
| *chico* ‘boy’ | *bastón* ‘cane’ | 0,41 |
| *chimpancé* ‘chimpanzee’ | *queso* ‘cheese’ | 0,29 |
| *doctor* ‘doctor’ | *caña de pescar* ‘fishing pole’ | 0,32 |
| *directora* ‘principal’ | fregona ‘mop’ | 0,3 |
| *empresaria* ‘business woman’ | *aguja de coser* ‘sewing needle’ | 0,67 |
| entrenador ‘coach’ | *coche* ‘car’ | 0,78 |
| *estudiante* ‘student’ | *tambor* ‘drum’ | 0,5 |
| *florista* ‘florist’ | *flecha* ‘arrow’ | 0,16 |
| *frutera* ‘green grocer’ | *gafas* ‘glasses’ | 0,69 |
| *gimnasta* ‘gymnast’ | *micrófono* ‘microphone’ | 0,14 |
| *hámster* ‘hamster’ | *hueso* ‘bone’ | 0,38 |
| *informático* ‘computer technician’ | *cruz* ‘cross’ | 0,25 |
| *jirafa* ‘giraffe’ | *jaula de pájaro* ‘bird cage’ | 0,07 |
| *lémur* ‘lemur’ | *biberón* ‘baby bottle’ | 0,23 |
| *lince* ‘lynx’ | *zanahoria* ‘carrot’ | 0,34 |
| *logopeda* ‘speech therapist’ | *camión de bomberos* ‘firetruck’ | 0,07 |
| *marmota* ‘groundhog’ | *huevo* ‘egg’ | 0,25 |
| *matemático* ‘mathematician’ | *tronco* ‘log’ | 0,21 |
| *modista* ‘dressmaker’ | *buzón* ‘mailbox’ | 0,2 |
| *paciente* ‘patient’ | *escoba* ‘broom’ | 0,12 |
| *periodista* ‘journalist’ | *madera* ‘wood’ | 0,21 |
| *pianista* ‘pianist’ | *bombilla* ‘light bulb’ | 0,29 |
| *pintor* ‘painter’ | *avión* ‘plane’ | 0,34 |
| *policía* ‘police officer’ | *peine* ‘comb’ | 0,45 |
| presentador ‘host’ | *barco* ‘ship’ | 0,21 |
| *psicólogo* ‘psychologist’ | *esposas* ‘handcuffs’ | 0,3 |
| *rehén* ‘hostage’ | *corona* ‘crown’ | 0,12 |
| *revisor* ‘reviser’ | *iglesia* ‘church’ | 0,21 |
| *taxista* ‘taxi driver’ | *violín* ‘violin’ | 0,2 |
| *viajero* ‘traveler’ | *máquina de escribir* ‘typewriter’ | 0,81 |

Online norming study results showing the mean ratings for weakly-related noun-noun pairs.
