# Supplementary material for: Subjects are not all alike: Eye-tracking the agent preference in Spanish
Source: PLoS One. 2022 Aug 3;17(8):e0272211. doi: 10.1371/journal.pone.0272211 (PMC9348668; doi:10.1371/journal.pone.0272211)
Supplement: S3 Table — Online norming study results showing the mean ratings for weakly-related verb-noun pairs. (DOCX) [file pone.0272211.s003.docx]

**S3 Table. Mean ratings of weakly-related verb-noun pairs.**

| **Noun** | **Verb** | **Mean rating** |
| --- | --- | --- |
| *anciano* ‘old man’ | *gritar* ‘shout’ | 1,72 |
| *anciano* ‘old man’ | *llegar* ‘arrive’ | 1 |
| *arquero* ‘archer’ | *aparecer* ‘appear’ | 0,54 |
| *arquero* ‘archer’ | *pasar* ‘go by’ | 0,4 |
| *asesino* ‘murderer’ | *llevar* ‘carry, wear’ | 0,69 |
| *atleta* ‘athlete’ | *matar* ‘kill’ | 0,09 |
| *barbero* ‘barber’ | *bailar* ‘dance’ | 0,27 |
| *barbero* ‘barber’ | *traer* ‘bring’ | 0,36 |
| *barrendero* ‘sweeper’ | *arreglar* ‘fix’ | 1,05 |
| *bebé* ‘baby’ | *arreglar* ‘fix’ | 0,2 |
| *bebé* ‘baby’ | *bailar* ‘dance’ | 0,87 |
| *bebé* ‘baby’ | *traer* ‘bring’ | 0,58 |
| *bombero* ‘firefighter’ | *girar* ‘turn’ | 0,8 |
| *bombero* ‘firefighter’ | *tocar* ‘play’ | 0,45 |
| *cachorro* ‘puppy’ | *morir* ‘die’ | 1,2 |
| *cachorro* ‘puppy’ | *saltar* ‘jump’ | 1,98 |
| *canario* ‘canary’ | *nadar* ‘swim’ | 0,12 |
| *canario* ‘canary’ | *pasar* ‘go by’ | 0,23 |
| *cantante* ‘singer’ | *limpiar* ‘clean’ | 0,18 |
| *cantante* ‘singer’ | *llamar* ‘call’ | 0,63 |
| *carpintero* ‘carpenter’ | *andar* ‘walk’ | 0,74 |
| *carpintero* ‘carpenter’ | *nacer* ‘be born’ | 0,63 |
| *cartero* ‘mailman’ | *comer* ‘eat’ | 1,05 |
| *cartero* ‘mailman’ | *contar* ‘tell, count’ | 1,6 |
| *chico* ‘boy’ | *gritar* ‘shout’ | 1,83 |
| *chico* ‘boy’ | *llegar* ‘arrive’ | 0,98 |
| *chimpancé* ‘chimpanzee’ | *caer* ‘fall’ | 0,87 |
| *chimpancé* ‘chimpanzee’ | *nacer* ‘be born’ | 1,52 |
| *científico* ‘scientist’ | *llevar* ‘carry, wear’ | 0,5 |
| *conductor* ‘driver’ | *pasear* ‘stroll’ | 1,6 |
| *conejo* ‘rabbit’ | *crecer* ‘grow’ | 1,67 |
| *conejo* ‘rabbit’ | *girar* ‘turn’ | 0,56 |
| *costurera* ‘seamstress’ | *correr* ‘run’ | 0,56 |
| *costurera* ‘seamstress’ | *desaparecer* ‘disappear’ | 0,25 |
| *doctor* ‘doctor’ | *limpiar* ‘clean’ | 1,29 |
| *doctor* ‘doctor’ | *morir* ‘die’ | 1,43 |
| *directora* ‘principal’ | *preparar* ‘prepare’ | 1,83 |
| *electricista* ‘electrician’ | *andar* ‘walk’ | 0,7 |
| *electricista* ‘electrician’ | *llevar’* carry, wear’ | 0,74 |
| *empresaria* ‘business woman’ | *correr* ‘run’ | 0,96 |
| *empresaria* ‘business woman’ | *desaparecer* ‘disappear’ | 0,47 |
| *entrenador* ‘coach’ | *pasear* ‘stroll’ | 1,2 |
| *escritor* ‘writer’ | *matar* ‘kill’ | 1,01 |
| *escritor* ‘writer’ | *nadar* ‘swim’ | 0,34 |
| *estudiante* ‘student’ | *preparar* ‘prepare’ | 1,32 |
| *explorador* ‘explorer’ | *matar* ‘kill’ | 0,47 |
| *florista* ‘florist’ | *aparecer* ‘appear’ | 0,32 |
| *florista* ‘florist’ | *pasar* ‘go by’ | 0,29 |
| *frutera* ‘green grocer’ | *pasear* ‘stroll’ | 0,7 |
| *frutera* ‘green grocer’ | *salir* ‘go out’ | 0,5 |
| *gallina* ‘hen’ | *correr* ‘run’ | 1,85 |
| *gallina* ‘hen’ | *desaparecer* ‘disappear’ | 0,49 |
| *gimnasta* ‘gymnast’ | *limpiar* ‘clean’ | 0,27 |
| *gimnasta* ‘gymnast’ | *llamar* ‘call’ | 0,4 |
| *hámster* ‘hamster’ | *caer* ‘fall’ | 0,69 |
| *hámster* ‘hamster’ | *caminar* ‘walk’ | 1,69 |
| *informático* ‘computer technician’ | *despertar* ‘wake up’ | 0,5 |
| *informático* ‘computer technician’ | *tocar* ‘touch’ | 0,9 |
| *jirafa* ‘giraffe’ | *nadar* ‘swim’ | 0,21 |
| *jirafa* ‘giraffe’ | *pasar* ‘go by’ | 0,49 |
| *lémur* ‘lemur’ | *arreglar* ‘fix’ | 0,03 |
| *leñador* ‘lumberjack’ | *gritar* ‘shout’ | 1,34 |
| *leñador* ‘lumberjack’ | *salir* ‘go out’ | 0,58 |
| *limpiadora* ‘cleaner’ | *preparar* ‘prepare’ | 1,5 |
| *lince* ‘lynx’ | *crecer* ‘grow’ | 1,76 |
| *lince* ‘lynx’ | *girar* ‘turn’ | 0,49 |
| *logopeda* ‘speech therapist’ | *girar* ‘turn’ | 0,2 |
| *logopeda* ‘speech therapist’ | *tocar* ‘touch’ | 0,43 |
| *marinero* ‘sailor’ | *aparecer* ‘appear’ | 0,96 |
| *marinero* ‘sailor’ | *caminar* ‘walk’ | 0,96 |
| *marmota* ‘groundhog’ | *correr* ‘run’ | 1,14 |
| *marmota* ‘groundhog’ | *desaparecer* ‘disappear’ | 0,63 |
| *matemático* ‘mathematician’ | *gritar* ‘shout’ | 0,54 |
| *matemático* ‘mathematician’ | *salir* ‘go out’ | 0,67 |
| *modista* ‘dressmaker’ | *comer* ‘eat’ | 1,18 |
| *modista* ‘dressmaker’ | *contar* ‘tell, count’ | 1,2 |
| *músico* ‘musician’ | *hablar* ‘speak’ | 1,61 |
| *músico* ‘musician’ | *llegar* ‘arrive’ | 0,9 |
| *oculista* ‘oculist’ | *pasear* ‘stroll’ | 0,58 |
| *oculista* ‘oculist’ | *salir* ‘go out’ | 0,52 |
| *paciente* ‘patient’ | *arreglar* ‘fix’ | 1,34 |
| *pájaro* ‘bird’ | *morir* ‘die’ | 1,49 |
| *pájaro* ‘bird’ | *saltar* ‘jump’ | 1,38 |
| *párroco* ‘priest’ | *llamar* ‘call’ | 1,52 |
| *peluquera* ‘hairdresser’ | *comer* ‘eat’ | 1,1 |
| *peluquera* ‘hairdresser’ | *contar* ‘tell, count’ | 1,43 |
| *percusionista* ‘drummer’ | *preparar* ‘prepare’ | 1,7 |
| *percusionista* ‘drummer’ | *andar* ‘walk’ | 1,49 |
| *periodista* ‘journalist’ | *nacer’* be born’ | 0,65 |
| *perro* ‘dog’ | *caer* ‘fall’ | 0,61 |
| *perro* ‘dog’ | *caminar* ‘walk’ | 1,65 |
| *pescador* ‘fisherman’ | *limpiar* ‘clean’ | 2 |
| *pescador* ‘fisherman’ | *morir* ‘die’ | 1,27 |
| *pianista* ‘pianist’ | *andar* ‘walk’ | 0,63 |
| *pianista* ‘pianist’ | *llevar* ‘carry, wear’ | 0,32 |
| *piloto* ‘pilot’ | *despertar* ‘wake up’ | 0,56 |
| *piloto* ‘pilot’ | *hablar* ‘speak’ | 1,78 |
| *pintor* ‘painter’ | *despertar* ‘wake up’ | 0,58 |
| *pintor* ‘painter’ | *hablar* ‘speak’ | 1,05 |
| *policía* ‘police officer’ | *comer* ‘eat’ | 1,47 |
| *policía* ‘police officer’ | *contar* ‘tell, count’ | 1,16 |
| *presentador* ‘host’ | *aparecer* ‘appear’ | 1,81 |
| *presentador* ‘host’ | *caminar* ‘walk’ | 1,07 |
| *preso* ‘inmate’ | *bailar* ‘dance’ | 0,54 |
| *preso* ‘inmate’ | *crecer* ‘grow’ | 0,52 |
| *psicólogo* ‘psychologist’ | *bailar* ‘dance’ | 0,74 |
| *psicólogo* ‘psychologist’ | *crecer* ‘grow’ | 1,74 |
| *ratón* ‘mouse’ | *caer* ‘fall’ | 0,43 |
| *ratón* ‘mouse’ | *nacer* ‘be born’ | 1,76 |
| *rehén* ‘hostage’ | *saltar* ‘jump’ | 1,1 |
| *rehén* ‘hostage’ | *traer* ‘bring’ | 0,83 |
| *reina* ‘queen’ | *saltar* ‘jump’ | 0,38 |
| *reina* ‘queen’ | *traer* ‘bring’ | 0,32 |
| *revisor* ‘reviser’ | *llamar* ‘call’ | 1,87 |
| *sacerdote* ‘priest’ | *despertar* ‘wake up’ | 0,4 |
| *sacerdote* ‘priest’ | *tocar* ‘touch’ | 1,09 |
| *taxista* ‘taxi driver’ | *hablar* ‘speak’ | 1,96 |
| *taxista* ‘taxi driver’ | *llegar* ‘arrive’ | 1,98 |
| *viajero* ‘traveler’ | *matar* ‘kill’ | 0,29 |

Online norming study results showing the mean ratings for weakly-related verb-noun pairs.
