# Supplementary material for: Subjects are not all alike: Eye-tracking the agent preference in Spanish
Source: PLoS One. 2022 Aug 3;17(8):e0272211. doi: 10.1371/journal.pone.0272211 (PMC9348668; doi:10.1371/journal.pone.0272211)
Supplement: Attachment — Submitted filename: Response to Reviewers.docx [file pone.0272211.s004.docx]

Vitoria-Gasteiz, May 26^th^, 2022

Dear Editor and Reviewers,

We are extremely thankful for your careful revision and enriching comments regarding our manuscript entitled “Subjects are not all alike: Eye-tracking the Agent Preference in Spanish.” We are particularly grateful for all the helpful ideas, suggestions and resources provided by the Reviewers, who analyzed our work thoroughly and kindly. After addressing each point raised by the Reviewers and applying the necessary changes, we hope that our revised manuscript will meet the criteria for publication in *PLOS ONE*.

Please find a detailed outline of our Response to the Reviewers below.

Sincerely,

Beatriz Gómez-Vidal

Pre-doctoral researcher

University of the Basque Country

Department of Linguistics and Basque Studies

+34 945014294

beatriz.gomezv@ehu.eus

**Review Comments to the Author**

Please find our Response to the Reviewers below.

**Points** raised by the Reviewers are numbered and marked in **blue bold Arial font**.

Responses are printed immediately below each point and marked in black Times New Roman font.

*Excerpts* from the first-submission or revised manuscript are left-indented and marked in *black Times New Roman font in italics*. Section, page number and line number are indicated.

**Reviewer 1**

1. **My biggest comment about the structure would be that the discussion seems a bit repetitive and could be cut by at 1 page.**

Response: We fully agree with this comment and have summarized the discussion accordingly, aiming to avoid any unnecessary repetitiveness. In the first-submission manuscript, the Discussion section covered 176 lines of text (pp. 24-30). In the revised manuscript, this section now covers 122 lines (pp. 25-30).

2. **In my opinion, there is only one major issue that needs to be addressed and it is speech rate.**

Response: We fully agree with this comment and thank the Reviewer for bringing it to our attention. We are also grateful for the Reviewer’s helpfulness in providing a useful reference (Fernández et al., 2020) to fix this problem and improve the quality of our research in the future. We have addressed the issue of speech rate thoroughly in our revised manuscript. Detailed responses to the concerns raised by the Reviewer on this issue can be found under points (3) and (4).

3. **Methods: If you went to the work to calculate the number of syllables of all items and length in msec then why not report it for the different conditions. And also, it is good practice to include the number of syllables per second for an accurate reporting of speech rate (see Fernandez et al. QJEP 2020). Speech rate is surprisingly consistent (within speaker) and so I wouldn’t expect condition differences to be a confound in your study. However, knowing the speech rate can be a key factor in the resolution of competing/mixed findings (e.g. Koring et al. (2012) vs. Huang & Snedeker (2020)).**

Response: We fully agree with this comment and have incorporated the necessary information to our revised manuscript. More specifically, we have included information on length (in syllables), duration (in milliseconds) and speech rate (in number of syllables per second) per condition in section 2.2 Stimuli. As for the previous papers that our work replicated (Koring et al., 2012; Huang & Snedeker, 2020), we have now included the fact that Koring et al. (2012) do not report speech rate in their study when addressing the issue of replicability in section 4.3 Global post-verbal frame. We have also addressed more clearly how speech rate could affect differences between conditions in our data. It should be noted that speech rate is not reported by Huang & Snedeker (2020) either.

*Section 2.2 Stimuli, page 9, lines 221-227: Experimental sentences had a mean length of 37 syllables (ranging from 31 to 46 syllables), a mean duration of 4244 ms and an average speech rate of 4.8 syllables per second (unaccusative condition: mean length 28 syllables, mean duration 5935 ms, average speech rate 4.6 syllables per second; unergative condition: mean length 29 syllables, mean duration 5829 ms, average speech rate 4.9 syllables per second; transitive condition: mean length 34 syllables, mean duration 6953 ms, average speech rate 4.9 syllables per second).*

*Section 4.3 Global post-verbal frame, page 21, lines 501-510: It could be the case that the pattern of increased and decreased fixations towards the visual target, which signals the reactivation and deactivation of the argument, was affected by experimental confounds. One such confound could be the speed of auditory presentation of the linguistic stimuli. Although our stimuli were identical to that of Koring et al. [19] in terms of syllabic length, the duration of the stimuli in milliseconds diverged between studies. Koring et al. [19] did not facilitate average speech rate in their study, but their linguistic stimuli lasted on average more than 4000 ms from sentential subject NP onset until verb offset. In our study, the equivalent linguistic stimuli lasted around 2000 ms. Hence, we concluded that our linguistic stimuli were presented at a faster speech rate than in Koring et al. [19], although our materials were recorded at a comfortable speaking rate.*

*Section 4.3 Global post-verbal frame, pages 21-22, lines 517-524: Second, faster speech rate could cause a delay in argument reactivation to a later time point than in Koring et al. [19] due to deferral in language processing [69]. Once again, this would not account for differences between conditions, since it would affect all conditions equally. To rule out the possibility that the peak in subject reactivation for the unaccusative condition was observed at a later point in our data, we conducted a post-hoc analysis including a wider time window, the global post-verbal frame, ranging from 200 ms to 3968 ms after verb offset (see section 3.2. for further details).*

4. **Results and Discussion: I agree with the speed of presentation issue, see comment above. However, I disagree with the conclusions on page 29. That is, that the peak should simply be later for a faster speech rate. In reality the effect of fast speech is a washing out of the linking between speech processing and direction of visual attention. BTW, the same also occurs for very slow speech. Again, I would highly recommend a detailed consultation of the Fernandez QJEP paper. The bottom line is that I think you need to (1) clearly report what your speech rate was, and (2) be very very clear in the discussion that speech rate should be factored in/considered in the interpretation. Is it possible to follow up on the H&S materials, were they fast or slow?**

Response: After consulting the suggested paper, we are in total agreement with the Reviewer and have changed the conclusions on this topic to the revised manuscript in section 5. Discussion. The other two comments raised by the Reviewer in point (4) have been discussed in our response to point (3) (see above).

*Section 5. Discussion, page 28, lines 658-663:* *At the global post-verbal time frame, the post-hoc analysis showed that agent subjects (unergative and transitive conditions) displayed a greater magnitude of reactivation than theme subjects (unaccusative condition). Once again, we found no evidence of a late peak in the reactivation of the unaccusative subject during this time frame. This indicates that the absence of a late peak in the reactivation of unaccusative subjects in our results was not due to a faster speech rate of the spoken sentences in our experiment.*

5. **There seems to be very little information about the fillers. I’d assume that some actually named objects shown in the array?**

Response: We have expanded the information about filler trials in the revised manuscript.

*Section 2.2 Stimuli, page 11, lines 267-271: The remaining trials were filler trials; in these, one of the words in the spoken sentence was directly matched by one of the drawings in the visual display. Filler trials were highly varied in their word order, syntactic structure and length of constituents. This was done in order to minimize the possibility of participants predicting the moment in which critical words would be presented in test and control trials.*

6. **The word derivation is used at several places in the introduction, but that word seems a bit misleading. Personally, I would tend to use derivation to refer to production. Because this study is comprehension, then shouldn’t the expected longer processing be because of the added interpretive steps with identifying the gap and assigning an argument to that gap.**

Response: We understand this concern raised by the Reviewer and have applied changes to the revised manuscript accordingly. More specifically, we have made a distinction between (a) the use of *derivation* when it pertains to works on theoretical linguistics, and (b) the use of *derivation* when it pertains to processing research, such as our own work. The word *derivation* has been maintained in those situations in which it is used by the original works that we cite, such as when describing the Unaccusative Hypothesis or other formal syntactic accounts. The word *derivation* has been substituted by other terms in those situations in which we talk about our own experiment and other processing research which does not use the term originally. In those cases, this word has been substituted by more appropriate terms such as “syntactic representation” or “interpretive steps”, like the Reviewer themselves suggested.

7. **Line 94: provide reference for reactivation of argument at trace.**

Response: We have incorporated references to the revised manuscript accordingly.

*Section 1.1 Unaccusativity and thematic roles, pages 3-4, lines 69-77: Studies using cross-modal lexical priming techniques [7,10,15] specifically explored whether unaccusative and unergative subjects were reactivated after the verb. Results showed a late post-verbal reactivation of the subject (around 750 ms after verb offset) in unaccusative predicates, but not in unergatives. According to the Trace Facilitation Hypothesis [7], speakers reactivate the mental representation of the referent at the point where its trace is encountered [16-18]. For this reason, their results [7,10,15] were interpreted as evidence that the unaccusative argument had deposited a trace within the VP due to syntactic movement, whereas the unergatives argument had not, as predicted by the UH.*

8. **Line 201: define test vs. control.**

Response: We have incorporated the necessary definition to the revised manuscript.

*Section 2.2 Stimuli, pages 8-9, lines 207-213: The present study had a 3 x 2 design with two independent variables: (i) verb type (unaccusative, unergative, transitive), and (ii) trial type (test, control). In test trials, the sentential subject shared a strong semantic relation with the target drawing (e.g., subject mouse, related to a depiction of a cheese), thus allowing us to measure subject reactivation. By contrast, in control trials the sentential subject did not share a strong semantic relation with the target drawing (e.g., subject chimpanzee, unrelated to a depiction of a cheese), thus serving as a baseline for fixations to the target drawing.*

9. **Line 216: report the comparisons of syllables in ROIs.**

Response: We have incorporated the average number of syllables for the relevant ROIs to the revised manuscript.

*Section 2.2 Stimuli, page 9, lines 232-235: Following Koring et al. [19], the number of syllables in ROIs 2 and 4 was thoroughly controlled to better align all experimental sentences at verb offset for the analysis. The average number of syllables in ROI 2 was 7.8, ranging from 7 to 9; the average number of syllables for ROI 4 was 13.8, ranging from 13 to 15.*

10. **Figure 4 should be more similar to others (axes, legend, etc.).**

Response: We agree with this comment and thank the Reviewer for bringing this inconsistency to our attention. We have generated Figure 4 again to match the others aesthetically in axes, legend and labels.

**Reviewer 2**

11. **The only major-ish comment is about the organization of the Introduction. If I understand correctly, most parts of the Introduction centered around the unaccusative hypothesis, whereas the agent first hypothesis was rarely discussed (the term was not even mentioned). Indeed, the second paragraph talked about the agent/theme distinction, but it seems a bit out of place and broke the flow of the writing. So I would suggest having a part that specifically introduces the theoretical motivation of the agent first hypothesis in the Introduction, such that the discussion of the two hypotheses can be more balanced.**

Response: We are in total agreement with this comment and thank the Reviewer for bringing this issue to our attention, since we believe that it has led to a significant improvement of our revised manuscript. Attending to this issue, we have reorganized the Introduction section in order to discuss both hypotheses equally. Most of the changes have been applied to section 1.1 Unaccusativity and thematic roles. In this section, first unaccusative and unergative predicates are introduced, together with the claims that theoretical linguists make about their syntactic representation and the thematic roles of their arguments. Then, the Unaccusative Hypothesis is discussed, followed by a quick summary of the sentence processing research that has tested this hypothesis experimentally. These works have found a processing distinction between unaccusative and unergative sentences, which they interpret as evidence of the Unaccusative Hypothesis. Next, the Agent First Hypothesis is introduced as an alternative explanation of said results, highlighting the fact this hypothesis is in accordance with other general findings in linguistic processing and cognitive research: that agents and themes are processed differently, with a greater preference for agents over themes. During this reorganization process, we have also summarized various parts of the Introduction in order to avoid repetitiveness, while also adding more information on the Agent First Hypothesis in order to discuss it more fully.

12. **Line 156. what is an "NVN" strategy? Please explain in the text.**

Response: We have incorporated an appropriate in-text definition to our revised manuscript.

*Section 1.2 Hypotheses, page 7, lines 163-165: The NVN strategy assumes a mapping of semantic roles to syntactic positions following an agent-action-patient order.*

13. **Line 208. A weird spacing.**

Response: We have deleted the extra spacing.

14. **Participants and procedure. I wonder how the listening-while-viewing task really worked. First of all, is there any cover story for the task? Did the participant have to do anything other than listen to the sentence and view the picture? Second, there was no fixation before the onset of the ROIs, so we have no idea what the status of eye-movement was at the onset of the ROIs. Would you please tell me whether the issues above might potentially influence the results of the experiment?**

Response: We agree with this comment and have incorporated more information on the procedure to our revised manuscript in section 2.3 Participants and procedure, as well as in section 3.1 Data processing, in order to address whether the issues raised by the Reviewer could influence the results of the experiment. The bottom line is that the validity of our results still stands, even if we did not check the status of the eye movements before the beginning of each trial. This is because the eye tracker captured the eye gaze effectively, which we checked during data processing. It is also because the location of the visual target was randomized on the screen, and thus it was impossible for participants to anticipate target location before the trial began. Finally, it is also because we used fixations on the visual target in control trials as a baseline for fixations to the target in test trials.

*Section 2.3 Participants and procedure, page 12, 302-312: Participants were told that they would hear some recorded sentences through the headphones while static visual displays showed on the screen. They were instructed to listen to the sentences very carefully while looking freely into the monitor. Participants were asked to fixate on a centrally-located cross that appeared between trials. This was done in order to reduce noise in the data, i.e., looks to a particular region of the screen before trial onset. Throughout the experiment, we did not check the status of the eye movements before sentence onset, but this did not pose a problem for our results due to (a) the randomization of the location of the visual target, (b) the use of control trials as a baseline for fixations in test trials. Participants did not have to perform any other task besides listening to the sentences and looking at the pictures. There was no cover story or recurring theme in the experiment.*

*Section 3.1 Data processing, page 13, lines 321-324: Data classified as “saccades”, “unclassified”, and instances of track loss, which represented 22.9% of the data, were eliminated from the dataset. This value indicated that collection of relevant data for our analysis was successful around 80% of the time.*

15. **Line 574-576. "In our study, we do not consider this prediction as one of the possible readings of the UH for eye-tracking data in the VWP". Are you referring to the results of your own experiment?**

Response: We have eliminated this comment from the revised manuscript. However, we have incorporated another comment much in the same vein to the Discussion in the revised manuscript. In any case, it is Koring et al. (2012) who claim that there is another possible reading of the UH for VWP data: one in which a greater magnitude of reactivation corresponds to greater processing cost. We argue that magnitude of activation or reactivation of an element in the VWP signals attention or preference, not processing cost.

*Section 5. Discussion, page 26, lines 603-606: Koring et al. [19] argue that the UH makes another prediction for eye-tracking data in the VWP: a larger reactivation of unaccusative subjects than unergatives, due to their greater processing cost of the former. Instead, we argue that the magnitude of argument reactivation is an indication of attention or preference, not of processing cost.*

16. **References. I think you need to provide the DOIs of the references.**

Response: We agree with this comment and thank the Reviewer for bringing this inconsistency to our attention. We have incorporated DOIs to the References section in the revised manuscript.
